# Climate change and its impact on mental health

**DOI:** 10.1007/s40211-025-00537-3

**Published:** 2025-08-04

**Authors:** Dinesh Bhugra

**Affiliations:** Kingʼs College, SE5 8AF London, UK

**Keywords:** Climate anxiety, Geopsychiatry, Eco-anxiety, Depression, Posttraumatic stress disorder, Klimaangst, Geo-Psychiatrie, Umweltangst, Depression, Posttraumatische Belastungsstörung

## Abstract

Climate change is the predominant global crisis of the 21st century and yet it appears as if not enough attention is being paid to its impact on health including mental health and wellbeing of populations globally. There is an increasing acknowledgement that eco-anxiety, solastalgia and other related conditions are emerging. However, more importantly the international impact of climate change with increasing internal and external migration places increasing strain on healthcare systems and healthcare professionals. The sheer speed of change related to climate factors started over 200 years ago but has accelerated in the past few decades and impacts human beings at multiple levels. Extreme weather events, rising temperatures, and environmental degradation contribute very strongly to both existing and newer psychiatric disorders. Recommendations are made for policymakers, researchers and clinicians about what is needed and how to deliver it.

## Abstract (de)

Der Klimawandel ist global die beherrschende Krise des 21. Jahrhunderts. Dennoch sieht es so aus, als werde seinem Einfluss auf die Gesundheit, einschließlich psychischer Gesundheit und Wohlbefinden der der Menschen auf der ganzen Welt, nicht genügend Aufmerksamkeit zuteil. Zunehmend wird anerkannt, dass sich Klimaangst, Solastalgie und weitere damit assoziierte Krankheitsbilder entwickeln. Noch wesentlich relevanter sind aber die internationalen Folgen des Klimawandels, wie zunehmende Binnenwanderungen und zwischenstaatliche Migrationsbewegungen, die Gesundheitssysteme und medizinisches Personal steigenden Belastungen aussetzen. Die rasante Geschwindigkeit der klimabedingten Veränderungen hat vor mehr als 200 Jahren begonnen, doch sie hat sich in den vergangenen Jahrzehnten beschleunigt und wirkt sich auf den Menschen auf mehreren Ebenen aus. Extreme Wetterereignisse, steigende Temperaturen und Umweltzerstörung tragen ganz erheblich zu bestehenden wie zu neueren psychiatrischen Beeinträchtigungen bei. Politischen Entscheidungsträgern sowie Forschenden und klinisch Tätigen werden Empfehlungen dazu gegeben, welche Maßnahmen erforderlich sind und wie sie sich realisieren lassen.

## Introduction

Although internationally climate change and its impact on human survival is increasingly being talked about with endless summits and agreements between politicians representing various countries but actions to deal with it seem to keep getting delayed or postponed on the flimsiest of excuses. There have been discussions about the impact of climate change on survival of humanity and also its effect on health, but as always mental health largely gets ignored although it appears that in the past few years this is beginning to change. In these circumstances, development of a new discipline within psychiatry—that of geopsychiatry—is particularly welcome. Geopsychiatry aims to look at geographical, political and commercial determinants and in turn their impact on social determinants which affect mental health and wellbeing. Furthermore, the field looks at wars, conflicts, natural and manmade disasters and their impact on mental health of individuals and communities. These disasters include climate change and its ongoing impact on mental health. These factors are international as often they occur across nations, whereas social determinants are more likely to be intranational.

Climate change is indeed a major health challenge across international and geographical borders but perhaps more importantly the speed of change is worrying to say the least. The impact of climate change occurs and is seen at different levels with varying consequences as illustrated later in this paper with profound implications for the future of psychiatric disorders. Research evidence indicates that extreme weather events, rising temperatures, and environmental degradation contribute very strongly to various psychiatric disorders. These include newer ones such as eco-anxiety, climate anxiety, solastalgia, etc. and older established ones such as depression, posttraumatic stress disorder, and suicide risk. Many vulnerable groups such as women, older adults, children and those with intellectual disability are further prone to developing climate change related psychiatric disorders. Climate change is not a homogenous phenomenon and includes various components such as air pollution, droughts, famines, flooding, etc., leading to increased migration within the country and across international borders. Climate change is leading to more frequent and extreme weather events which can have a traumatic impact on populations. It is worth noting that not everyone will be affected in the same way. People are affected in multiple ways and with specific support systems may respond in different ways so the healthcare professionals must be sensitive to that.

There is an urgent pressing need to look at the impact of climate change on different levels but before we do that it would be helpful to be clear about certain definitions.

## Definitions

According to the United Nations (not dated) climate change refers to long-term shifts in temperatures and weather patterns. As the document notes, these shifts and changes can be natural, due to changes in the sun’s activity or large volcanic eruptions. Over the past two centuries and more, this change is directly attributable to human activity primarily due to the burning of fossil fuels like coal, oil and gas [1]. Burning fossil fuels generates greenhouse gas emissions that trap the sun’s heat, raising temperatures. IPCC [1] reports that the main greenhouse gases causing climate change include carbon dioxide and methane. There are multiple sources of these from using gasoline for driving a car, air travel, burning coal for heating buildings, and methane produced by animals such as cows. In addition, destruction of forests, contribution of forest fires, etc. can further add to the greenhouse effect. Energy, industry, transport, buildings, agriculture and land use are among other factors that can contribute to greenhouse gases.

## Climate events

The impact of climate change on health including mental health and public health is important to be recognized and clearly depends upon type of event. Some are acute events and then there are ongoing chronic activities such as air pollution. There is a growing body of robust scientific evidence which confirms that global warming and extreme weather events affect health (including mental health) and such effects can be short-, medium- and long-term and these effects can be profound. However, in terms of any actions, certainly there is much left to be desired.

Palinkas and Wong [2] classify three types of climate-related events which have an impact on mental health and wellbeing. These include the following: (1) acute events such as hurricanes, tsunamis, typhoons, floods, and wildfires; (2) subacute or long-term changes such as drought and heat stress; and (3) the existential threat of long-lasting changes, which may lead to higher temperatures, rising sea levels and a permanently altered and potentially uninhabitable physical environment. The impact of these events on health can be both direct (i.e., heat stress) and indirect (i.e., economic loss, threats to health and wellbeing, displacement and forced migration, collective violence and civil conflict, and alienation from a degraded environment) consequences of global climate change.

**Fig. 1:**
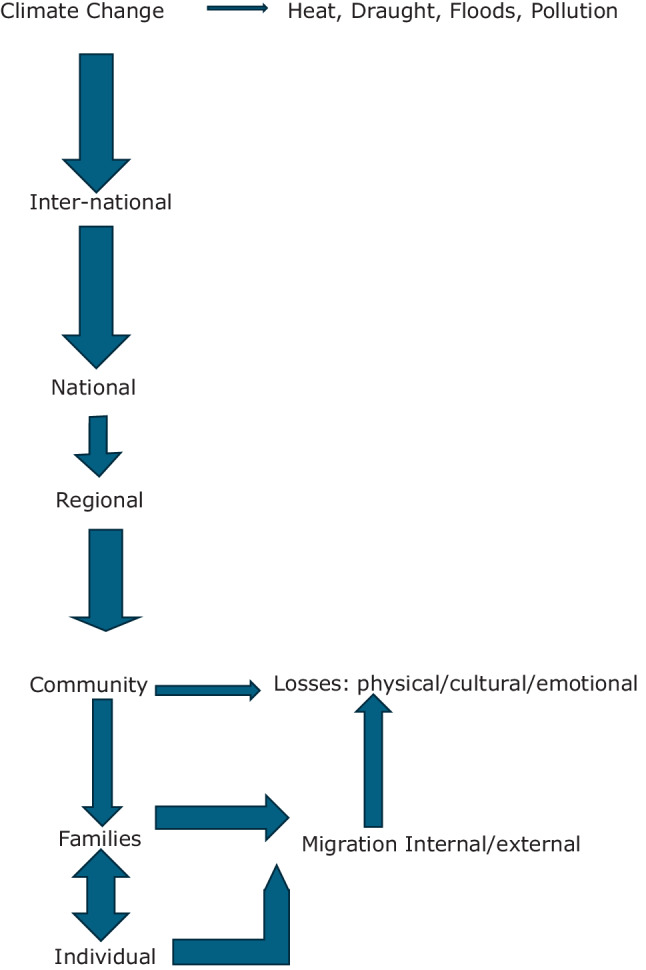
Impact of climate change at various levels

Severe weather events—such as floods, droughts, wildfires, and heatwaves—are significantly associated with increased prevalence of certain psychiatric conditions as mentioned above. Climate change impacts at various levels: these include at individual, family, community, local, regional, national and international levels. And the response at each level will be different and possibly complex. These consequences can be caused by but also lead to forced involuntary acts of migration, loss of status, stress, psychiatric disorders, etc, which further add to loss of emotional stability, sense of belonging, and the psychological wellbeing of entire communities (see Figure 1). In this context, climate change emerges as a salient contributor to what has been described as “polycrisis.” This situation reflects a convergence of interdependent crises that collectively undermine human capabilities, long-term prospects, and overall wellbeing. This further brings complexity to an already complex field where socio-psycho-bio-spiritual-anthropological factors contribute to etiology of various psychiatric disorders as well as therapeutic interventions. Various authors have argued persuasively for prioritizing preventive and adaptive interventions [3–5]. These factors are likely to have major and serious impact on design and delivery of psychiatric services.

In a systematic review, Walinski et al. [6] studied both the direct effects of acute extreme weather events (floods, storms, fires) and chronic stresses (heat, drought) due to climate change, and the indirect effects of climate change (food insecurity, migration), on the diagnoses of mental disorders, psychological distress, and psychiatric emergency admissions. Not surprisingly, they found that heterogeneity of studies and data were problematic, but they concluded that the traumatic experiences due to extreme weather events increase the risk of affective and anxiety disorders, especially the risk of posttraumatic stress disorder. This also seemed related to heat (high temperatures) which further increased the morbidity and mortality attributable to mental illnesses and psychiatric emergencies. In addition, continuing environmental challenges related to draughts, food insecurity and consequent migration contributed to the likelihood of higher rates of psychiatric disorders.

In a scoping review, Charlsson et al. [7] also reported that several climate-related exposures, including heat, humidity, rainfall, drought, wildfires, and floods were associated with psychological distress, worsened mental health. They also found that these changes were associated with higher mortality among people with pre-existing mental health conditions, increased psychiatric hospitalizations, and heightened suicide rates.

## Impact

IPCC [8] have noted that virtually all global heating over the last 200 years largely has been due to greenhouse gases that are warming the world faster than at any time in at least the last two thousand years. WMO [9] has indicated that the temperatures are higher than befor, with the last decade being the warmest on record [9]. However, it needs emphasizing that temperature rise is only one aspect of climate change because its causes and consequences are both complex and important in our understanding what is happening to the planet as a system. The systems approach is important because of the interconnectedness of countries, environments, trade, climate, etc. and thus the slightest change in one area can influence changes in all others leading to the aphorism that when a butterfly flaps its wings in one part of the world, typhoons can occur in another part. Intense droughts, water scarcity, forest fires, rise in sea levels, flooding, melting icebergs, storms, tsunamis are all interconnected but quite often focus is on or the other rather than in an interconnected way.

The World Health Organization (WHO) [10] has estimated that between 2030 and 2050, climate change is expected to cause approximately 250,000 additional deaths per year, from undernutrition, malaria, diarrhea and heat stress alone. WHO reckons that the direct damage costs to health (excluding costs in health-determining sectors such as agriculture and water and sanitation) is estimated to be between US $ 2–4 billion per year by 2030. We know that health including mental health is interlinked with housing, employment, education, food, etc. and as climate change will affect ability to grow food, housing, safety and work, rates of various psychiatric disorders are likely to rise. With rising sea levels there is an imminent danger that many island nations such as Maldives, Barbados and other places may well submerge under water. On the other hand, many countries in the global south may be vulnerable to water scarcity and droughts. These conditions are likely to push people into migrating internally as well as across borders.

The emissions that cause climate change come from every part of the world and affect everyone. However, countries such as the USA and emerging markets such as China, Brazil and India are high polluters [11]. Action points recommended by the United Nations (UN) and various agreements such as Paris agreement will not be repeated here. Suffice it to say without being unduly alarmist and pessimistic, that unless drastic measures are taken to cut emissions, the situation does look bleak. There are relatively accessible solutions such as switching to clean energy and other appropriate sustainability measures provided there is political will. Complicating factors include lobbyists on behalf of interested parties from commercial sector, armaments, fossil fuel industries, etc.

Li et al. [4] caution that the climate and ecological crisis will constitute the defining public health challenge of the twenty-first century. This inevitably will include impact on mental health as physical and mental illnesses affect each other in a variety of ways. Thus, there is an unprecedented global threat to all determinants of health, and consequently to healthcare delivery systems with increasing demands and strain on the healthcare system.

## Mental health

As mentioned above, there can be direct and indirect impacts on mental health of individuals, families and communities. Extreme weather events, like typhoons, tsunamis, hurricanes and floods, can cause psychological distress and trauma through loss of material possessions but also loss of support and bereavement. Furthermore, the impact can be on pre-existing conditions as well as developing new ones. In addition, survivor guilt may be seen in vulnerable individuals. Actual events as well as expected events may cause anxiety, depression, and suicidal ideation and acts of suicide. Air pollution and infectious diseases, which can be exacerbated by climate change, also have direct and indirect impacts on mental health and wellbeing of individuals. Newer categories of clinical diagnoses such as eco-anxiety, climate anxiety, eco-grief, solastalgia, etc. are beginning to emerge but need very careful study and evaluation.

At a broader level, such extreme weather events will affect determinants such as unemployment, loss of housing, food and water insecurity and other social consequences. Bonnano et al. [12] reported that most people following an extreme weather event can recover fully but it is worth being emphasizing that repeated events may affect subsequent ability to cope. Increasing temperatures and heat can lead to various psychiatric disorders as well as worsen the mental state of individuals with pre-existing psychiatric conditions. Burke et al. [13] demonstrated that rates of suicide increased when the temperatures went up. The cause and effect need further exploration although heat can produce sleep disturbances and negative thinking. Romanello et al. [14] reported variations across countries with a larger than expected effect in low-income countries. Another complicating factor that must be borne in mind is that many psychiatric medications can disrupt the person’s ability to regulate body temperature and contribute to sleep disturbances during heat waves.

### Air pollution

Climate change and air pollution are related, these add to the amount of allergens and pollutants which can affect breathing and short-term exposure has been shown to have higher risk of psychiatric admissions [15]. Newbury et al. [16] reported that levels of air pollution are linked with increased service use by individuals with existing psychotic disorders.

### Infectious diseases

It has been argued that due to climate change and heat, there is also a risk of increase in infectious diseases such as dengue, malaria, zika, etc. [17]. These are likely to influence mental state leading to bereavement, anxiety, depression, PTSD, survivor guilt and whole range of other issues which affect physical and mental health. Pihkala [15] described climate anxiety and other emerging psychological reactions to climate change such as solastalgia defined as the inability of finding solace in a familiar landscape due to environmental degradation. Ecological grief is about the sense of loss emerging from experiencing environmental degradation and climate anxiety (also termed as eco-anxiety which is anxiety in the face of climate change). These symptoms can contribute to poor sleep [18].

There is no doubt that the impact of climate change is differential across countries and even within the same country. Hickman et al. [19] in a survey of young people across countries found that a significant majority of young people were moderately or severely worried about climate change and were disappointed with government responses in their country.

## What is needed?

Three areas of policy, research and clinical services require attention. Some of the recommendations are listed below.

### Policy and practice changes

It is perhaps not entirely surprising that in the WHO survey of 95 countries only 9 included mental health and psychosocial support in their national health and climate change plans.

- The new WHO policy brief recommends 5 important approaches for governments to address the mental health impacts of climate change. These include integration of mental health programs and support with climate change discussions. Furthermore, working across borders is a must. Working with communities to build resilience and reduce vulnerabilities with appropriate and adequate funding.
- Policy makers must have a mental health impact assessment of all the polices especially those related to climate change.
- Policymakers must make transparent decisions in discussion with stakeholders.
- A clear evaluation of resources needed must be identified and funded as a matter of urgency.
- Research and clinical services must be funded adequately.
- Sustainability at all levels, in all institutions must be the norm.
- Disaster preparation must have flexibilities to scale up interventions as needed.
- Public mental health is important and education using multimedia approaches can be very helpful. As Li et al. [4] propose development of climate cafés which provide a safe space to focus on impact of climate where people can get together regularly and share their thoughts, experiences and mutual learning while working with vulnerable individuals.

### Research

- Researchers must develop tools for measuring the wide-ranging impact of climate change on individual, community and population mental health.
- Data scientists, climate scientists and climate-attribution researchers, geographers, political economists, anthropologists, and sociologists must come together with mental health researchers to understand the impact of climate change on wellbeing of populations.
- Researchers must engage with communities and vulnerable groups and indigenous communities to focus on what is needed and developing research questions accordingly.
- There needs to be an urgent attempt to focus on low- and middle-income countries who are likely to face the most impact of climate change.
- Stigma related to mental illnesses varies across cultures and needs careful study in the face of climate change and its consequences.
- Developing agreed diagnostic criteria in partnership with clinicians is incredibly important.
- Researchers must develop and track standardized ways to measure milder or more fleeting forms of eco-anxiety and distress that fall outside standard diagnoses but also global indicators to establish types of interventions.

### Clinicians

- Health professionals including mental health professionals have a moral responsibility to advance public understanding of the climate crisis by highlighting its impact on physical and mental wellbeing, and advocating for systemic changes to limit its impending harms.
- Mental-health professionals also need training and support to provide help. They also require help in learning about sustainability of services as well as advocacy.
- They should form partnerships with community organizations and other stakeholders to develop and deliver public health programs.
- Prevention and public mental health are the key and such approaches can lead to building resilience especially among vulnerable groups.
- Supporting individuals in understanding and taking appropriate action can help mitigate eco-anxiety.
- There may be situations where community involvement can help reduce anxiety among those who are worried, angry or scared, whereas others may require specialist interventions. Clinicians need to be aware of different levels of anxiety and interventions.
- Working with nongovernmental organizations (NGOs) can be helpful. Advocacy, local involvement in sustainability programs need developing further wherever possible.

## Conclusion

The consequences of climate change are stress factors for mental health. Therefore, as global warming progresses, an increasing incidence and prevalence of mental illness is to be expected. Vulnerable groups, such as the (already) mentally ill, children, and adolescents, need to be protected. There is a need for further systematic research on the mechanisms of action and effects of climate change on mental function.

In view of the urgency of the impact of climate change and its consequences on the mental health and wellbeing of individuals, there is a pressing need to adapt psychiatric training, fortify healthcare infrastructure, and implement climate-responsive policies. Telepsychiatry and other digital interventions may be helpful in urgent assessments as well as ensuring continuity of care. Research on neurobiological stress markers may offer innovative pathways for prevention and early intervention. Psychiatry must play an active role in climate change mitigation and adaptation efforts, advocating for climate justice and equitable access to mental health services. Addressing the psychiatric dimensions of climate change is essential for building resilient mental health systems in the decades ahead.
